# Association of oxidative balance score with metabolic syndrome and its components in middle-aged and older individuals in the United States

**DOI:** 10.3389/fnut.2025.1523791

**Published:** 2025-02-06

**Authors:** Qu Zhang, Yemei Wu, Bo Luo

**Affiliations:** ^1^Department of Radiotherapy Center, Breast Cancer Center, National Key Clinical Specialty Construction Discipline, Hubei Provincial Clinical Research Center for Breast Cancer, Wuhan Clinical Research Center for Breast Cancer, Tongji Medical College, Hubei Cancer Hospital, Huazhong University of Science and Technology, Wuhan, China; ^2^Tongji Medical College, Hubei Cancer Hospital, Huazhong University of Science and Technology, Wuhan, China

**Keywords:** metabolic syndrome, oxidative balance score, middle-aged and older individuals, National Health and Nutrition Examination Survey, dietary and lifestyle factors

## Abstract

**Background:**

The prevalence of metabolic syndrome (MetS) among middle-aged and older individuals in the U.S. is rising, posing significant mortality risks. Diet is a key factor in MetS development, yet few studies have examined the combined effects of dietary and lifestyle factors on MetS in this group. Recently, the oxidative balance score (OBS), an indicator of oxidative status encompassing diet and physical activity, has attracted interest. This study explores the association between OBS and MetS, as well as its individual components, in middle-aged and older Americans.

**Methods:**

Data from 6,157 participants aged 45 years and older in the National Health and Nutrition Examination Survey (NHANES) (1999–2018) were analyzed. The OBS was calculated using 16 dietary and four lifestyle factors. Logistic regression was used to assess associations between OBS and MetS. Separate analyses examined dietary OBS (DOBS) and lifestyle OBS (LOBS) in relation to MetS.

**Results:**

Higher OBS quartiles were associated with a reduced MetS risk (OR 0.25; 95% confidence interval [CI]: 0.12–0.51; *p* < 0.0001), after adjusting for confounders. Increased OBS was linked to decreases in waist circumference (WC) (OR 0.41; 95% CI: 0.30–0.51; *p* < 0.0001), triglycerides (TG) (OR 0.71; 95% CI: 0.53–0.92; *p* = 0.0139), blood pressure (BP) (OR 0.53; 95% CI: 0.40–0.69; *p* < 0.0001), and fasting glucose (FG) (OR 0.61; 95% CI: 0.45–0.81; *p* < 0.0001), while HDL-C increased (OR 0.68; 95% CI: 0.51–0.90; *p* = 0.0065). DOBS was inversely associated with MetS through reductions in BP and FG and increased HDL-C, though it showed no significant effect on WC or TG. LOBS was associated with reductions across WC, BP, FG, TG, and an increase in HDL-C.

**Conclusion:**

OBS is inversely associated with MetS in middle-aged and older U.S. adults. Enhancing OBS through dietary guidelines emphasizing antioxidant-rich foods, fiber, and unsaturated fats, alongside lifestyle changes like regular exercise, smoking cessation, and moderate alcohol intake, may be crucial in MetS prevention for this population.

## 1 Introduction

Metabolic Syndrome (MetS) is a cluster of multiple metabolic abnormalities, including central obesity, hypertension, hyperglycemia, and dyslipidemia (1). It is a significant risk factor for cardiovascular disease and diabetes. With rapid economic development and lifestyle changes, the prevalence of MetS has been rising globally (2), especially among middle-aged and older populations. While numerous studies have explored the relationship between diet and MetS (3), there is relatively limited research on the combined effects of dietary and lifestyle factors on MetS, particularly in the context of oxidative balance.

The Oxidative Balance Score (OBS) is a novel indicator that integrates dietary and physical activity factors to assess the balance between antioxidants and pro-oxidants in the body. Research has shown that a higher OBS is associated with reduced risks of various chronic diseases. For instance, a study based on the National Health and Nutrition Examination Survey (NHANES) database analyzed 10,591 participants with diabetes and prediabetes, finding an inverse relationship between OBS and risks of all-cause and cardiovascular mortality. Each unit increase in OBS was associated with a 1.8% reduction in all-cause mortality risk (hazard ratio [HR] 0.982, 95% confidence interval [CI] 0.976–0.987) and a 4% reduction in cardiovascular mortality risk (HR 0.960, 95% CI 0.949–0.970), highlighting the importance of an antioxidant-rich diet and lifestyle for individuals with diabetes and prediabetes (4). Another study, based on NHANES data from 2011 to 2018, investigated the association between OBS and obesity/body composition in young and middle-aged adults. It found that a higher OBS was associated with a lower likelihood of obesity (as defined by BMI, OR = 0.43, 95% CI = 0.36–0.50) and a lower fat mass percentage (FM %). Moreover, each unit increase in OBS was inversely associated with FM% across various body regions and positively associated with lean mass percentage (LM %), emphasizing the potential role of antioxidant diets and lifestyle interventions in preventing obesity and improving body composition (5). Additionally, a prospective cohort study from the Korean Genome and Epidemiology Study (KoGES) examined the relationship between OBS and incident hypertension (HTN) in adults. It found that a higher OBS was associated with a reduced risk of developing hypertension, with this effect observed in both men and women. This underscores the potential value of improving oxidative balance in preventing hypertension (6). A cross-sectional study involving 26,118 adults also demonstrated an inverse association between OBS and dyslipidemia, highlighting the protective role of antioxidant-rich diets against lipid abnormalities (7).

Previous studies have also shown that the OBS is inversely associated with the risk of MetS and its components. An analysis based on NHANES data from 2011 to 2018 found that a higher OBS was linked to a lower risk of MetS, and the OBS was inversely associated with the risks of all five components of MetS (high triglycerides, low HDL cholesterol, elevated fasting glucose, high waist circumference, and hypertension). The relationship between OBS and MetS or its components was found to be nonlinear. These findings suggest that OBS may serve as an effective marker for identifying individuals with MetS, with a higher OBS indicating greater antioxidant activity, which may help prevent MetS and its related health issues (8). Another NHANES study showed that a higher OBS was associated with lower levels of oxidative stress and an inverse relationship with the risk of MetS and its components, emphasizing the potential role of antioxidant-rich diets and lifestyles in preventing MetS (9). Finally, a study involving 11,171 adult participants from NHANES confirmed the inverse association between a higher OBS and reduced MetS risk, lower MetS severity, and decreased all-cause mortality, further supporting the importance of antioxidants in the prevention and management of MetS (10).

However, despite these studies providing preliminary evidence for the role of the OBS in MetS, the relationship between OBS and MetS in middle-aged and older populations in the United States has not been adequately explored. This study aims to fill this gap by analyzing a nationally representative sample of U.S. adults aged 45 years and older to investigate the associations between OBS and MetS, as well as its individual components. Our objective is to provide evidence for developing targeted prevention strategies to reduce health risks associated with MetS.

## 2 Materials and methods

The nutritional and health statuses of the U.S. population were evaluated through a cross-sectional study known as the NHANES, which is accessible at: https://www.cdc.gov/nchs/nhanes/index.htm. Every year, NHANES collects data from approximately 5,000 individuals and new datasets are released biennially. The participants did not overlap between the different survey cycles. The survey entailed questionnaires, physical examinations, and laboratory tests that provided comprehensive data on socioeconomic, demographic, dietary, health-related, exercise, and lifestyle variables. Ethical approval for this research was granted by the Research Ethics Review Board of the National Center for Health Statistics,^1^ and all participants provided written consent prior to participation. This study was conducted per the principles of the Declaration of Helsinki.

### 2.1 Inclusion criteria

**Figure 1:**
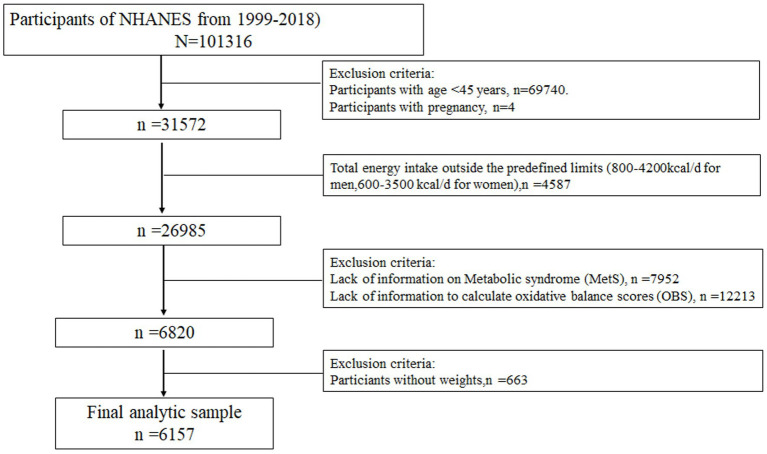
Flow chart of the study population.

Descriptive data from 10 consecutive NHANES cycles, covering 1999–2018, were analyzed in this study. According to the definition provided by the China Health and Retirement Longitudinal Study (CHARLS), middle-aged and older adults are defined as individuals aged 45 years and above (11). Information on demographics, obesity status, blood pressure, blood glucose levels, lipid profiles, diet, lifestyle behaviors, comorbidities, and other health-related factors were collected. Of the initial 101,316 participants recruited, 69,740 were younger than 45 years and excluded. Additionally, 4,587 participants with unrealistic energy intake values (daily caloric intake ranging from 800 to 4,200 kcal for men and 600–3,500 kcal for women) were excluded. Only participants with complete OBS and metabolic syndrome data (*n* = 20,165) were included. Finally, 663 individuals with missing weight-related data were excluded, resulting in a final analytical sample of 6,157 participants (Figure 1).

### 2.2 Oxidative balance scores

The study determined participants’ OBS using a method previously employed in research (12, 13). This approach evaluated 16 dietary elements and four lifestyle factors influencing oxidative stress (14). The components are categorized into: (1) nutritional antioxidants, including fiber, beta-carotene, riboflavin, niacin, vitamin B6, folate, vitamin B12, vitamin C, vitamin E, calcium, magnesium, zinc, copper, and selenium; (2) dietary factors promoting oxidation, including total fat intake and iron levels; (3) physical activity, which serves as a source of lifestyle-related antioxidants; and (4) lifestyle factors that enhance oxidative stress, including alcohol intake, smoking behaviors, and body mass index (BMI).

Dietary elements were tiered based on their distribution among the participants, with antioxidants scoring from 0–2 across tertiles and pro-oxidants scored inversely. For lifestyle metrics, physical activity was scored as follows: <150 min/week scored 0, 150–300 min/week scored 1, and > 300 min/week scored 2. Regarding alcohol intake, women consuming <12 drinks/year scored 2, whereas those consuming <1 drink daily scored 1. Men consuming <2 drinks daily scored 1 and those consuming ≥2 drinks daily scored 0. Smoking was assessed using cotinine levels, with scoring inverse to the distribution, from 0 (highest tertile) to 2 (lowest tertile). BMI categories assigned points as follows: normal weight (<25 kg/m^2^) scored 1, overweight (25–29.9 kg/m^2^) scored 2, and obese (≥30 kg/m^2^) scored 0. Overall, OBS ranged from 0 to 40 and was computed from all aggregated scores. Furthermore, dietary and lifestyle OBS were computed by summing the scores from the dietary and lifestyle components, respectively. Higher scores indicate greater antioxidant exposure. The scoring scheme for OBS components is detailed in Supplementary Table S1.

### 2.3 Definition of metabolic syndrome

MetS was diagnosed following the 2005 NCEP guidelines (2). MetS was defined based on the fulfillment of three or more predefined criteria: (1) Elevated WC, defined as >88 cm for women and > 102 cm for men; (2) Elevated TG, characterized by TG levels >1.7 mmol/L (150 mg/dL) or current use of TG-lowering medications; (3) Elevated BP, defined as >130/85 mmHg or use of antihypertensive medication; (4) Elevated FG, defined as fasting glucose >5.6 mmol/L (100 mg/dL) or use of glucose-lowering medication; (5) Reduced HDL-C, defined as <1.3 mmol/L (50 mg/dL) for women or < 1.0 mmol/L (40 mg/dL) for men, or use of lipid-lowering medication.

### 2.4 Covariates

The analysis included the following covariates: age, race/ethnicity (Non-Hispanic Black, Non-Hispanic White, Hispanic, and others), educational level, family socioeconomic status, and marital status (categorized as currently married or living with a partner or others). Education was classified into four levels: less than high school, high school or equivalent, college without a bachelor’s degree, and bachelor’s degree or above. Family socioeconomic status was determined by the family poverty income ratio, with low-income status highlighted as a key category.

### 2.5 Statistical analysis

All analyses accounted for sample weights to reflect the complex structure of the NHANES. Participants’ baseline characteristics were summarized, with continuous variables presented as means accompanied by standard deviations (SDs) and categorical variables reported as counts (n) along with their corresponding percentages (%). OBS was evaluated both as a continuous variable and by dividing it into tertiles, with the lowest tertile (tertile 1) designated as the reference group. Multivariate logistic regression analyses were conducted to estimate the odds ratios (ORs) and their corresponding 95% confidence intervals (CIs) to investigate the potential relationship between OBS and MetS risk. Two models were developed: Model 1, which was unadjusted, and Model 2, which was adjusted for various covariates (age, sex, race/ethnicity, educational attainment, marital status, family income-to-poverty ratio, health insurance coverage, and total energy intake). Collinearity assessments confirmed that multicollinearity was not a concern in Model 2. Restricted cubic splines with a smoothing function were used to investigate the association in a dose-dependent manner. Additionally, the relationships between DOBS, LOBS, and MetS risk were analyzed. Multivariate linear regression analyses were performed to evaluate the relationship between OBS and MetS. All statistical analyses were performed using R version 4.2.1 (R Foundation for Statistical Computing, Vienna, Austria) and SAS 9.4 software (SAS Institute Inc., Cary, NC, USA), with statistical significance set at *p* < 0.05.

## 3 Results

### 3.1 Distribution of OBS tertile features among study participants

The characteristics of study participants, grouped by OBS tertiles, are displayed in Table 1. Among the 6,157 NHANES participants (mean age: 60.47 years), 2,941 (47.77%) were men and 3,216 (52.23%) were women. Individuals in the highest OBS tertile were younger, Non-Hispanic White, married, and had a lower MetS prevalence, lower educational levels, higher poverty-to-income ratios, and greater energy drink and caffeine intake. Furthermore, individuals in the highest OBS tertile demonstrated lower systolic blood pressure (BPXSY), diastolic blood pressure (BPXDI), triglyceride levels, fasting glucose levels, and waist circumference, with elevated levels of HDL-C, compared with participants in the lowest OBS tertile. A negative correlation was identified between elevated OBS and the probability of developing MetS. Table 1 presents the comprehensive baseline characteristics stratified according to OBS tertiles.

**Table 1:** The baseline characteristics by tertiles of the OBS: National Health and Nutrition Examination Survey 1999–2018 (NHANES 1999–2018).^1^

Characteristics	Total (6,157)	Tertile 1	Tertile 2	Tertile 3	*p*-value
Age (years)	60.47 (0.23)	61.25 (0.44)	60.90 (0.36)	59.58 (0.32)	0.0045
PIR	3.21 (0.05)	2.52 (0.07)	3.23 (0.06)	3.65 (0.07)	<0.0001
Energy intake (kcal/day)	1991.01 (13.61)	1473.05 (17.94)	1950.25 (24.19)	2365.00 (21.84)	<0.0001
Metabolic syndrome					<0.0001
Non-Mets	359 (7.05)	52 (2.92)	114 (5.59)	193 (11.07)	
MetS	5,798 (92.95)	1897 (97.08)	2002 (94.41)	1899 (88.93)	
Caffeine (mg)	195.00 (3.96)	180.49 (7.28)	198.63 (6.65)	201.13 (6.24)	0.0129
BPXSY	126.20 (0.40)	129.55 (0.79)	127.10 (0.56)	123.19 (0.52)	<0.0001
BPXDI	70.05 (0.31)	69.72 (0.53)	70.21 (0.46)	70.11 (0.45)	0.5834
TG	1.48 (0.02)	1.58 (0.04)	1.49 (0.04)	1.40 (0.03)	<0.0001
Glu	6.25 (0.05)	6.50 (0.08)	6.28 (0.07)	6.06 (0.05)	<0.0001
HDL-C	1.43 (0.01)	1.38 (0.02)	1.42 (0.02)	1.47 (0.01)	<0.0001
WC	102.04 (0.41)	104.45 (0.50)	103.13 (0.70)	99.46 (0.53)	<0.0001
Marital status, Married (n, %)	3,844 (67.52)	1,099 (58.36)	1,345 (68.91)	1,400 (72.20)	<0.0001
Educational level (n, %)					<0.0001
College or above	1,490 (24.20)	667 (25.83)	488 (14.17)	335 (9.32)	
High school or equivalent	1,436 (23.32)	524 (30.38)	528 (28.04)	384 (16.97)	
Less than high school	3,231 (52.48)	758 (43.78)	1,100 (57.79)	1,373 (73.71)	
Race (*n*, %)					<0.0001
Non-Hispanic White	3,005 (74.51)	854 (67.62)	1,053 (75.01)	1,098 (78.53)	
Non-Hispanic Black	1,212 (8.87)	550 (15.31)	386 (8.14)	276 (5.36)	
Mexian American	760 (5.40)	223 (5.85)	261 (5.70)	276 (4.84)	
Others	1,180 (11.22)	322 (11.23)	416 (11.15)	442 (11.27)	

^1^All estimates accounted for complex survey designs in NHANES. Values were mean ± standard error for continuous variables and numbers (percentages) for categorical variables. MetS, Metabolic syndrome; OBS, oxidative balance score; PIR, family income-to-poverty ratio; BPXSY, systolic blood pressure; BPXDI, diastolic blood pressure; TG, total triglycerides; Glu, blood sugar; HDL-C, high-density lipoprotein; WC, Waist Circumference (cm).

Considering the sex-specific OBS construction differences, we further analyzed the baseline characteristics separately for men and women, as shown in Tables S2 and S3. These results are consistent with those observed in the overall population. In women, no significant relationship was found between caffeine consumption and BPXDI (Supplementary Table S2). In men, MetS, BPXDI, HDL-C level, and caffeine intake were not significant (Supplementary Table S3).

### 3.2 Association of OBS with MetS

**Figure 2:**
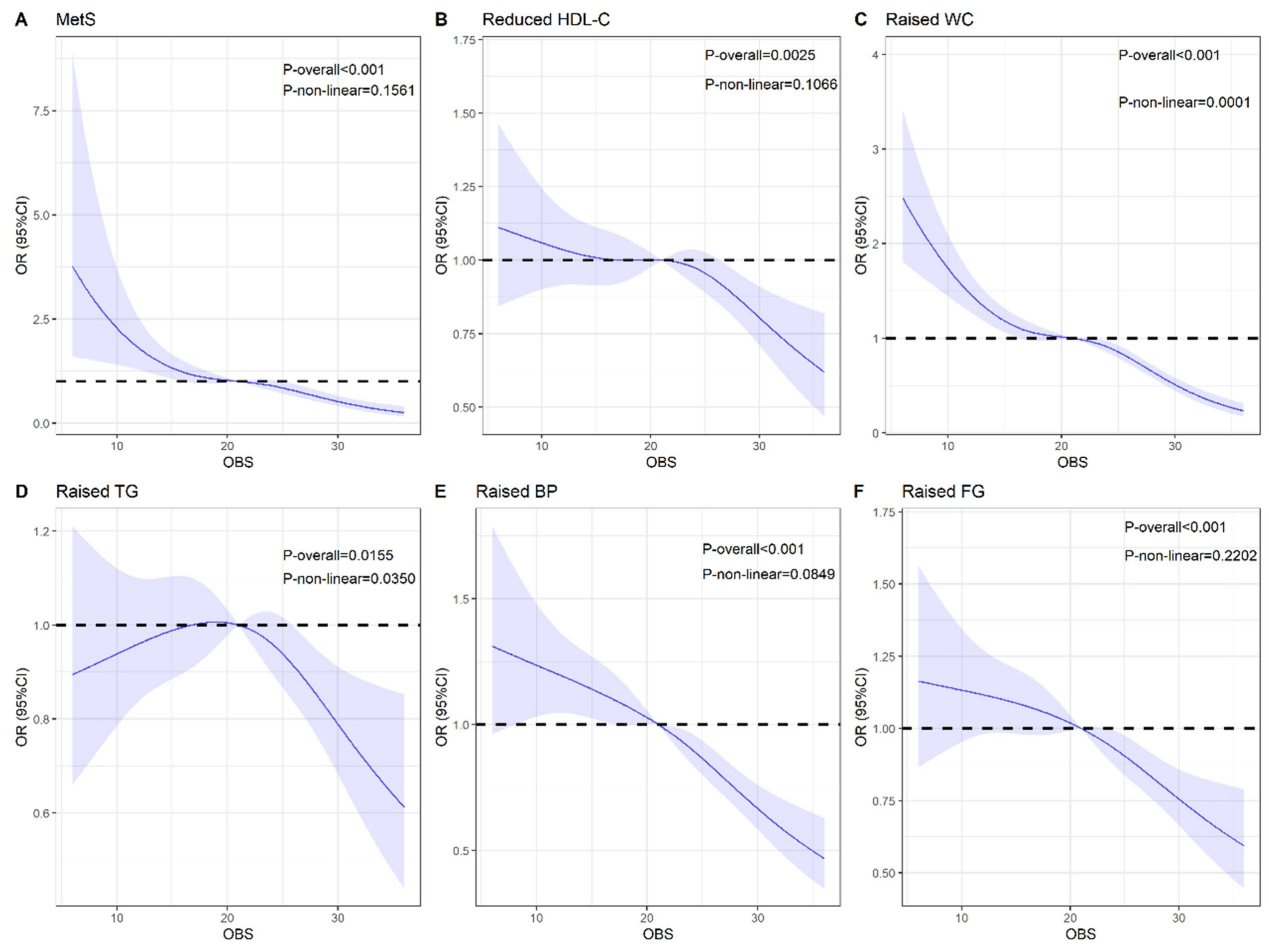
The restricted cubic spline for the associations of OBS with MetS and its components. **(A)** Metabolic syndrome; **(B)** Reduced HDL-C; **(C)** Raised WC; **(D)** Raised TG; **(E)** Raised BP; **(F)** Raised FG. All estimates accounted for complex survey designs in NHANES. MetS, Metabolic syndrome; OBS, oxidative balance score; OR, odds ratio; CI, confidence interval. WC, waist circumference; FG, fasting blood glucose; HDL-C, cholesterol, TG, blood triglycerides; BP, blood pressure.

To investigate the relationship between OBS and MetS incidence, we conducted a binary logistic regression analysis along with its constituent components (Table 2). After considering sociodemographic characteristics and behavioral variables, individuals in the highest OBS tertile (Tertile 3) exhibited a 75% reduced risk of developing MetS compared with those in the lowest tertile (Tertile 1), with an OR of 0.25 (95% CI: 0.12–0.51, *p* < 0.0001). When analyzing the individual MetS components, a higher OBS was significantly associated with reduced waist circumference (OR: 0.41, 95% CI: 0.30–0.57, *p* < 0.0001), triglycerides (OR: 0.71, 95% CI: 0.53–0.92, *p* = 0.0139), blood pressure (OR: 0.53, 95% CI: 0.40–0.69, *p* < 0.0001), and fasting glucose (OR: 0.61, 95% CI: 0.45–0.81, *p* < 0.0001), and with higher HDL-C levels (OR: 0.68, 95% CI: 0.51–0.90, *p* = 0.0065). Furthermore, spline regression models revealed a significant decrease in MetS risk and its individual components as OBS increased (Figure 2).

**Table 2:** The relationship between OBS and MetS in National Health and Nutrition Examination Survey 1999–2018 (NHANES 1999–2018).^1^

Mets (OR 95%CI)	Unadjusted OR (95%CI)	Adjusted OR (95%CI)
Tertile 1	1.00 (reference)	1.00 (reference)
Tertile 2	0.51 (0.29–0.90)	0.47 (0.24–0.94)
Tertile 3	0.24 (0.15–0.40)	0.25 (0.12–0.51)
*P* trend	<0.0001	<0.0001
Raised WC (OR 95% CI)		
Tertile 1	1.00 (reference)	1.00 (reference)
Tertile 2	0.78 (0.62–1.00)	0.64 (0.49–0.85)
Tertile 3	0.59 (0.46–0.75)	0.41 (0.30–0.57)
*P* trend	<0.0001	<0.0001
Raised TG (OR 95% CI)		
Tertile 1	1.00 (reference)	1.00 (reference)
Tertile 2	0.73 (0.58–0.92)	0.76 (0.60–0.96)
Tertile 3	0.65 (0.51–0.83)	0.71 (0.53–0.92)
*P* trend	0.0008	0.0139
Reduced HDL-C (OR 95% CI)		
Tertile 1	1.00 (reference)	1.00 (reference)
Tertile 2	0.81 (0.66–1.01)	0.84 (0.66–1.06)
Tertile 3	0.63 (0.51–0.78)	0.68 (0.51–0.90)
*P* trend	<0.0001	0.0065
Raised BP (OR 95% CI)		
Tertile 1	1.00 (reference)	1.00 (reference)
Tertile 2	0.79 (0.63–0.98)	0.80 (0.62–1.03)
Tertile 3	0.51 (0.41–0.62)	0.53 (0.40–0.69)
*P* trend	<0.0001	<0.0001
raise FG (OR 95% CI)		
Tertile 1	1.00 (reference)	1.00 (reference)
Tertile 2	0.77 (0.64–0.91)	0.76 (0.62–0.94)
Tertile 3	0.57 (0.47–0.69)	0.61 (0.45–0.81)
*P* trend	<0.0001	0.001

^1All estimates accounted for complex survey designs in NHANES. 2Model 1: unadjusted. 3Model 2: adjusted for age, sex, family income-to-poverty ratio, marital status and educational level. 4Model 3: adjusted for all variables in model 1 and further for caffeine intake, energy intake and history of comorbidities. MetS, Metabolic syndrome; OBS, oxidative balance score; OR, odds ratio; CI, confidence interval; WC, waist circumference; FG, fasting blood glucose; HDL-C, cholesterol, TG, blood triglycerides; BP, blood pressure.^

### 3.3 Subgroup analysis

To investigate the potential effects of sex, we performed a sex-stratified analysis (Table 3). After adjusting for other variables, the results indicated that in women, a higher OBS score was significantly associated with reduced MetS prevalence, smaller WC, lower BP and FG levels, and higher HDL-C levels. Conversely, in men, the OBS was not significantly associated with overall MetS but was significantly related to elevated WC and hypertension. As OBS increased, the risk of elevated WC and hypertension decreased in men. We performed stratified subgroup analyses based on race, educational attainment, age, marital status, and poverty-to-income ratio in addition to sex-specific analysis (Supplementary Table S4), with significant variations noted solely regarding sex.

**Table 3:** The relationship between OBS and MetS in National Health and Nutrition Examination Survey 1999–2018 (NHANES 1999–2018) ^1^.

Tertile of OBS	Female		Male	
	Model 1^2^	Model 2^3^	Model 1^2^	Model 2^3^
MetS (OR 95% CI)				
Tertile 1	1.00 (reference)	1.00 (reference)	1.00 (reference)	1.00 (reference)
Tertile 2	0.29 (0.15–0.58)	0.25 (0.11–0.56)	0.75 (0.35–1.62)	0.82 (0.34–1.94)
Tertile 3	0.11 (0.06–0.19)	0.11 (0.05–0.24)	0.52 (0.25–1.10)	0.59 (0.22–1.62)
*P* trend	<0.0001	<0.0001	0.0715	0.2669
Raised WC (OR 95% CI)				
Tertile 1	1.00 (reference)	1.00 (reference)	1.00 (reference)	1.00 (reference)
Tertile 2	0.75 (0.54–1.05)	0.64 (0.42–0.96)	0.52 (0.25–1.10)	0.67 (0.47–0.94)
Tertile 3	0.49 (0.36–0.68)	0.39 (0.24–0.62)	0.65 (0.47–0.90)	0.46 (0.29–0.72)
*P* trend	<0.0001	<0.0001	0.012	0.0012
Raised TG (OR 95% CI)				
Tertile 1	1.00 (reference)	1.00 (reference)	1.00 (reference)	1.00 (reference)
Tertile 2	0.66 (0.48–0.90)	0.60 (0.45–0.82)	0.82 (0.58–1.18)	0.83 (0.54–1.28)
Tertile 3	0.54 (0.38–0.77)	0.50 (0.32–0.78)	0.80 (0.55–1.16)	0.83 (0.52–1.33)
*P* trend	0.0009	0.0039	0.2585	0.4796
Reduced HDL-C (OR 95% CI)				
Tertile 1	1.00 (reference)	1.00 (reference)	1.00 (reference)	1.00 (reference)
Tertile 2	0.77 (0.61–0.99)	0.81 (0.60–1.10)	0.86 (0.61–1.21)	0.85 (0.60–1.22)
Tertile 3	0.50 (0.38–0.65)	0.57 (0.39–0.84)	0.83 (0.62–1.13)	0.81 (0.56–1.17)
*P* trend	<0.0001	0.0043	0.2573	0.2878
Raised BP (OR 95% CI)				
Tertile 1	1.00 (reference)	1.00 (reference)	1.00 (reference)	1.00 (reference)
Tertile 2	0.77 (0.58–1.02)	0.78 (0.55–1.09)	0.81 (0.58–1.12)	0.88 (0.59–1.13)
Tertile 3	0.47 (0.36–0.62)	0.52 (0.37–0.74)	0.55 (0.42–0.73)	0.62 (0.41–0.93)
*P* trend	<0.0001	0.0004	<0.0001	0.014
raise FG (OR 95% CI)				
Tertile 1	1.00 (reference)	1.00 (reference)	1.00 (reference)	1.00 (reference)
Tertile 2	0.66 (0.50–0.88)	0.66 (0.48–0.89)	0.91 (0.67–1.24)	0.92 (0.65–1.29)
Tertile 3	0.47 (0.36–0.63)	0.50 (0.35–0.71)	0.73 (0.51–1.03)	0.74 (0.47–1.16)
*P*-value	<0.0001	0.0002	0.0754	0.1837

^1All estimates accounted for complex survey designs in NHANES. 2Model 1: unadjusted. 3Model 2: adjusted for age, sex, family income-to-poverty ratio, marital status educational level, caffeine intake, energy intake, and history of comorbidities. MetS, Metabolic syndrome; OBS, oxidative balance score; OR, odds ratio; CI, confidence interval. WC, waist circumference; FG, fasting blood glucose; HDL-C, cholesterol, TG, blood triglycerides; BP, blood pressure.^

### 3.4 Association of DOBS and LOBS with MetS

The OBS was further categorized into DOBS and LOBS; their individual effects on MetS are detailed in Supplementary Table S5. The findings revealed that DOBS was associated with a decreased likelihood of developing MetS, particularly in subgroups characterized by elevated TG, HDL-C, and BP, where significant associations were observed. Likewise, higher LOBS showed a significant correlation with a reduced MetS risk, smaller WC, lower BP, reduced FG, and increased HDL-C levels.

## 4 Discussion

This study utilized data from the NHANES to reveal a negative association between the OBS and MetS in middle-aged and older adults. Our results indicate that a higher OBS is associated with a lower risk of MetS in this population, emphasizing the effectiveness of the OBS as an indicator for assessing the impact of diet and lifestyle on metabolic health.

Our study further elucidates the relationship between the OBS and components of MetS. Specifically, a higher OBS was linked to lower waist circumference, blood pressure, fasting glucose levels, and higher high-density lipoprotein cholesterol (HDL-C) levels. The potential mechanisms include: (1) OBS and fasting glucose (FG): Oxidative stress exacerbates insulin resistance and increases blood glucose by activating inflammatory signaling pathways (such as NF-κB) and reducing insulin sensitivity (15, 16). Higher OBS may inhibit free radical production through increased dietary antioxidant intake (e.g., vitamins C and E), improving insulin signaling and lowering blood glucose levels (17). Oxidative stress can also induce pancreatic *β*-cell apoptosis (18), and antioxidant intake may protect these cells’ functions (19). (2) OBS and blood pressure (BP): Oxidative stress impairs endothelial function and reduces nitric oxide (NO) bioavailability, leading to vasoconstriction and hypertension (20). Higher antioxidant intake may reduce hypertension risk by improving endothelial function and decreasing angiotensin II production (21). (3) OBS and lipid profile (triglycerides (TG) and HDL-C): Lipid peroxidation increases the oxidation of triglycerides and low-density lipoproteins (LDL). Improving OBS may reduce lipid peroxidation, thereby enhancing HDL-C levels (12). A higher OBS is also associated with the intake of unsaturated fatty acids in dietary patterns (22), which may regulate liver lipid metabolism enzymes to lower TG levels and increase HDL-C. (4) OBS and waist circumference (WC): Obesity, especially the accumulation of visceral fat, is closely related to chronic oxidative stress (23). OBS may improve fat metabolism by reducing pro-oxidative dietary components and pro-inflammatory factors, thereby decreasing visceral fat (24).

Sex differences are another important finding in this study. In women, the association between the OBS and MetS and its components (such as waist circumference, blood pressure, fasting glucose, and HDL-C) was more significant. In contrast, in men, the OBS was not significantly correlated with overall MetS but was associated with specific components, such as waist circumference and hypertension. These differences may be related to physiological (25), hormonal (26), and lifestyle factors (27) that influence the relationship between OBS and metabolic health indicators (28).

Antioxidant-rich dietary patterns, such as the DASH diet and Mediterranean diet, have been shown to have positive effects on metabolic health (29). These dietary patterns, rich in fruits, vegetables, whole grains, and healthy fats, are closely associated with improved metabolic health (30). This study further confirms the critical role of dietary antioxidants in reducing oxidative stress and inflammation. Their potential mechanisms include neutralizing free radicals and reducing the damage caused by reactive oxygen species to cell membranes, DNA, and proteins, thereby lowering the risk of diseases associated with metabolic syndrome (31, 32). Antioxidants may also enhance the cell’s antioxidant defense capacity by activating the Nrf2 signaling pathway (33).

Furthermore, this study found that lifestyle-related OBS was more strongly negatively correlated with MetS compared to dietary-related OBS. This suggests that, in addition to diet, positive lifestyle factors, such as regular physical activity and moderate alcohol consumption, are crucial for reducing oxidative stress levels and improving insulin sensitivity. These factors may have a more significant impact on increasing the OBS and lowering MetS risk (34). Combining a healthy diet with regular physical activity may lead to more pronounced benefits.

Therefore, this study suggests increasing the intake of antioxidant-rich foods, such as dark leafy vegetables, nuts, and olive oil, to improve the OBS, while reducing the intake of pro-oxidants, such as high-fat and high-iron diets. Regular moderate-intensity aerobic exercise, such as walking and swimming, is recommended to improve lifestyle-related OBS. For specific high-risk populations, such as middle-aged and older adults and individuals with obesity, integrated intervention programs combining diet and exercise should be designed to improve the OBS.

Although this study, based on NHANES data, provides a large sample size and comprehensive dietary and lifestyle information, there are still some limitations. As a cross-sectional study, it cannot determine the causal relationship between OBS and MetS, and future prospective cohort studies will help verify these findings. Additionally, although we controlled for multiple confounding factors, residual confounding due to unmeasured variables may still exist. The dietary data collected through 24-h dietary recall may not accurately reflect long-term eating habits and could be subject to recall bias. This study used the oxidative balance scoring system and did not directly measure plasma antioxidant levels or inflammatory markers, so future studies should incorporate biochemical data to further validate the relationship between OBS and metabolic health. Lastly, since this study was conducted in the U.S. population, the relationship between OBS and MetS may vary in different cultural contexts. Future research should expand to other populations for validation.

## Data Availability

The study’s data originated from the National Health and Nutrition Examination Survey (NHANES), which can be accessed via https://www.cdc.gov/nchs/nhanes/index.htm.

## Footnotes

^1^ https://www.cdc.gov/nchs/nhanes/irba98.htm
