# Supplementary material for: Association of oxidative balance score with metabolic syndrome and its components in middle-aged and older individuals in the United States
Source: Front Nutr. 2025 Feb 6;12:1523791. doi: 10.3389/fnut.2025.1523791 (PMC11839438; doi:10.3389/fnut.2025.1523791)
Supplement: Supplementary file 1 [file Table_1.DOCX]

Table S1. Oxidative balance score assignment scheme.

| OBS component | Property | Male | | | Female | | |
| --- | --- | --- | --- | --- | --- | --- | --- |
|  |  | 0 | 1 | 2 | 0 | 1 | 2 |
| Dietary OBS components |  |  |  |  |  |  |  |
| Calcium(mg/d) | A | <702.50 | 702.50-1052.50 | ≥1052.50 | ＜595.50 | 595.50-879.00 | ≥879.00 |
| Carotene(RE/d) | A | ＜728.00 | 728.00-2576.50 | ≥2576.50 | ＜815.00 | 815.00-2850.00 | ≥2850.00 |
| Copper (mg/d) | A | ＜1.00 | 1.00-1.41 | ≥1.41 | ＜0.85 | 0.85-1.19 | ≥1.19 |
| Total fat (g/d) | A | ＜65.05 | 65.05-95.26 | ≥95.26 | ＜49.40 | 49.40-72.18 | ≥72.18 |
| Dietary fiber (g/d) | A | ＜13.30 | 13.30-20.95 | ≥20.95 | ＜11.40 | 11.40-17.40 | ≥17.40 |
| Iron(mg/d) | A | ＜12.16 | 12.16-17.68 | ≥17.68 | ＜9.55 | 9.55-13.54 | ≥13.54 |
| Magnesium (mg/d) | A | ＜250.50 | 250.50-350.00 | ≥350.00 | ＜206.50 | 206.50-286.00 | ≥286.00 |
| Niacin (mg/d) | A | ＜21.48 | 21.48-29.98 | ≥29.98 | ＜15.55 | 15.55-21.94 | ≥21.94 |
| Riboflavin (mg/d) | A | ＜1.75 | 1.75-2.47 | ≥2.47 | ＜1.38 | 1.38-1.93 | ≥1.93 |
| Selenium(mcg/d) | A | ＜97.25 | 97.25-135.40 | ≥135.40 | ＜70.65 | 70.65-101.20 | ≥101.20 |
| Total folate (mcg/d) | A | ＜351.50 | 351.50-474.00 | ≥474.00 | ＜253.50 | 253.50-373.50 | ≥373.50 |
| Vitamin B6 (mg/d) | A | ＜1.67 | 1.67-2.43 | ≥2.43 | ＜1.28 | 1.28-1.85 | ≥1.85 |
| Vitamin B12 (mcg/d) | A | ＜3.41 | 3.41-5.82 | ≥5.82 | ＜2.53 | 2.53-4.26 | ≥4.26 |
| Vitamin C (mg/d) | A | ＜42.90 | 42.90-99.25 | ≥99.25 | ＜41.65 | 41.65-90.65 | ≥90.65 |
| Vitamin E (ATE) (mg/d) | A | ＜5.93 | 5.93-9.16 | ≥9.16 | ＜4.89 | 4.89-7.83 | ≥7.83 |
| Zinc (mg/d) | A | ＜9.25 | 9.25-13.28 | ≥13.28 | ＜6.99 | 6.99-9.92 | ≥9.92 |
| Lifestyle OBS components |  |  |  |  |  |  |  |
| Physical  (MET-minute/week) | A | ＜400.00 | 400.00-2640.00 | ≥2640.00 | ＜40.00 | 40.00-1200.00 | ≥1200.00 |
| Cotinine (ng/ml) | P | ＜0.02 | 0.02-0.20 | ≥0.20 | ＜0.02 | 0.02-0.06 | ≥0.06 |
| Alcohol (g/d) | P | ≥30.00 | 0-30.00 | None | ≥15.00 | 0.00-15.00 | None |
| Body mass index (kg/m^2^) | A | ＜26.22 | 26.22-30.49 | ≥30.49 | ＜26.24 | 26.24-31.79 | ≥31.79 |

A stood for the antioxidant, P for the pro-oxidant, RE for the retinal equivalent, ATE for the alpha-tocopherol equivalent, and MET for the metabolic equivalent.

Table S2. The baseline characteristics of females by tertiles of the OBS: National Health and Nutrition Examination Survey 1999–2018 (NHANES 1999–2018)^1^.

| Characteristics | Total (3216) | Tertile 1 | Tertile 2 | Tertile 3 | *P* value |
| --- | --- | --- | --- | --- | --- |
| Age (years) | 60.77 (0.29) | 61.37 (0.56) | 61.74 (0.46) | 59.52 (0.41) | 0.037 |
| PIR | 3.11 (0.06) | 2.40 (0.08) | 3.04 (0.08) | 3.62 (0.08) | <.0001 |
| Energy intake (kcal/day) | 1725.69 (17.28) | 1259.01 (16.89) | 1698.16 (27.50) | 2050.75 (23.98) | <.0001 |
| Metabolic syndrome |  |  |  |  | <.0001 |
| Non-Mets | 189 (7.60) | 19 (1.61) | 54 (5.36) | 116 (13.45) |  |
| MetS | 3027 (92.40) | 989 (98.39) | 1040 (94.64) | 998 (86.55) |  |
| Caffeine (mg) | 170.03 (4.80) | 151.90 (8.36) | 175.78 (8.23) | 176.60 (7.65) | 0.0508 |
| BPXSY | 125.95 (0.47) | 129.19 (0.96) | 127.79 (0.76) | 122.24 (0.71) | <.0001 |
| BPXDI | 68.65 (0.35) | 67.79 (0.63) | 69.09 (0.49) | 68.82 (0.53) | 0.4205 |
| TG | 1.41 (0.02) | 1.56 (0.04) | 1.42 (0.04) | 1.31 (0.04) | <.0001 |
| Glu | 6.04 (0.04) | 6.37 (0.07) | 5.99 (0.06) | 5.88 (0.06) | <.0001 |
| HDL-C | 1.57 (0.01) | 1.49 (0.02) | 1.56 (0.02) | 1.62 (0.02) | <.0001 |
| WC | 99.29 (0.48) | 101.79 (0.77) | 100.70 (0.83) | 96.42 (0.64) | <.0001 |
| Marital status, Married (n, %) | 1707 (61.29) | 471 (51.15) | 576 (62.23) | 660 (66.99) | <.0001 |
| Educational level (n, %) |  |  |  |  | <.0001 |
| College or above | 762 (23.69) | 342 (26.78) | 251 (14.35) | 169 (9.48) |  |
| High school or equivalent | 771 (23.97) | 282 (33.08) | 288 (28.61) | 201 (16.10) |  |
| Less than high school | 1683 (52.33) | 384 (40.14) | 555 (57.04) | 744 (74.42) |  |
| Race (n, %) |  |  |  |  | <.0001 |
| Non-Hispanic White | 1539 (73.55) | 422 (65.26) | 543 (74.56) | 574 (78.00) |  |
| Non-Hispanic Black | 656 (9.63) | 294 (17.11) | 202 (8.56) | 160 (5.76) |  |
| Mexian American | 404 (5.40) | 122 (5.20) | 137 (6.13) | 145 (4.89) |  |
| Others | 617 (11.42) | 170 (12.43) | 212 (10.76) | 235 (11.35) |  |

^1^ All estimates accounted for complex survey designs in NHANES. Values were mean ± standard error for continuous variables and numbers (percentages) for categorical variables. Abbreviation and acronyms: MetS, Metabolic syndrome; OBS, oxidative balance score; PIR family income-to-poverty ratio. BPXSY: systolic blood pressure; BPXDI: diastolic blood pressure; TG: total triglycerides; Glu: blood sugar; HDL-C: high-density lipoprotein; WC: Waist Circumference (cm)

Table S3: The baseline characteristics of males by tertiles of the OBS: National Health and Nutrition Examination Survey 1999–2018 (NHANES 1999–2018)^1^.

| Characteristics | Total (2941) | Tertile 1 | Tertile 2 | Tertile 3 | *P* value |
| --- | --- | --- | --- | --- | --- |
| Age (years) | 60.13 (0.29) | 61.13 (0.57) | 59.94 (0.44) | 59.65 (0.39) | 0.0181 |
| PIR | 3.34 (0.06) | 2.66 (0.09) | 3.45 (0.08) | 3.68 (0.08) | <.0001 |
| Energy intake (kcal/day) | 2301.15 (19.09) | 1725.30 (24.58) | 2234.35 (33.35) | 2742.81 (29.12) | <.0001 |
| Metabolic syndrome |  |  |  |  | 0.1692 |
| Non-Mets | 170(6.405) | 33(4.4549) | 60(5.861) | 77(8.2016) |  |
| MetS | 2771(93.595) | 908(95.5451) | 962(94.139) | 901(91.7984) |  |
| Caffeine (mg) | 224.20 (6.60) | 214.18 (11.72) | 224.37 (11.00) | 230.62 (10.58) | 0.1713 |
| BPXSY | 126.49 (0.57) | 129.97 (1.24) | 126.33 (0.73) | 124.35 (0.82) | 0.0011 |
| BPXDI | 71.68 (0.39) | 71.99 (0.72) | 71.48 (0.61) | 71.66 (0.60) | 0.9315 |
| TG | 1.55 (0.04) | 1.61 (0.07) | 1.56 (0.07) | 1.50 (0.06) | 0.0411 |
| Glu | 6.50 (0.07) | 6.65 (0.13) | 6.62 (0.12) | 6.28 (0.09) | 0.0149 |
| HDL-C | 1.27 (0.01) | 1.25 (0.02) | 1.27 (0.02) | 1.29 (0.02) | 0.1456 |
| WC | 105.25 (0.54) | 107.59 (0.67) | 105.88 (0.92) | 103.11 (0.85) | <.0001 |
| Marital status, Married (n, %) | 2137 (74.80) | 628 (66.86) | 769 (76.44) | 740 (78.45) | 0.0031 |
| Educational level (n, %) |  |  |  |  | <.0001 |
| College or above | 728 (24.75) | 325 (24.72) | 237 (13.96) | 166 (9.14) |  |
| High school or equivalent | 665 (22.61) | 242 (27.21) | 240 (27.40) | 183 (18.01) |  |
| Less than high school | 1548 (52.64) | 374 (48.07) | 545 (58.64) | 629 (72.85) |  |
| Race (n, %) |  |  |  |  | <.0001 |
| Non-Hispanic White | 1466 (75.63) | 432 (70.39) | 510 (75.53) | 524 (79.16) |  |
| Non-Hispanic Black | 556 (7.99) | 256 (13.18) | 184 (7.67) | 116 (4.87) |  |
| Mexian American | 356 (5.40) | 101 (6.61) | 124 (5.21) | 131 (4.79) |  |
| Others | 563 (10.98) | 152 (9.82) | 204 (11.59) | 207 (11.18) |  |

^1^ All estimates accounted for complex survey designs in NHANES. Values were mean ± standard error for continuous variables and numbers (percentages) for categorical variables. Abbreviation and acronyms: MetS,Metabolic syndrome;OBS, oxidative balance score; PIR family income-to-poverty ratio. BPXSY: systolic blood pressure; BPXDI: diastolic blood pressure; TG：total triglycerides; Glu: blood sugar; HDL-C:high-density lipoprotein; WC:Waist Circumference (cm)

Table S4. Stratified analysis of associations of OBS with Mets in US adult population, NHANES 1999–2018^1^.

| Stratified factors |  | MetS (OR 95%CI) | *P* for interaction |
| --- | --- | --- | --- |
| **Sex** |  |  | 0.0092 |
|  | Tertile 1 | 1.00 (reference) |  |
| Male | Tertile 2 | 0.83 (0.35 - 1.95) |  |
|  | Tertile 3 | 0.61 (0.23 - 1.62) |  |
|  | Tertile 1 | 1.00 (reference) |  |
| Female | Tertile 2 | 0.24 (0.11 - 0.53) |  |
|  | Tertile 3 | 0.11 (0.05 - 0.23) |  |
| **Age (years)** |  |  | 0.1804 |
|  | Tertile 1 | 1.00 (reference) |  |
| Age < 65 | Tertile 2 | 0.47 (0.36 - 0.62) |  |
|  | Tertile 3 | 0.36 (0.27 - 0.48) |  |
|  | Tertile 1 | 1.00 (reference) |  |
| Age ≥ 65 | Tertile 2 | 1.59 (0.60 -4.2) |  |
|  | Tertile 3 | 0.35(0.14 -0.88) |  |
| **Race** |  |  | 0.3486 |
|  | Tertile 1 | 1.00 (reference) |  |
| Non-Hispanic White | Tertile 2 | 0.85 (0.43 - 1.67) |  |
|  | Tertile 3 | 0.38 (0.18 - 0.80) |  |
|  | Tertile 1 | 1.00 (reference) |  |
| Others | Tertile 2 | 0.38 (0.15 - 0.98) |  |
|  | Tertile 3 | 0.22 (0.08 - 0.57) |  |
| **Education** |  |  | 0.2681 |
|  | Tertile 1 | 1.00 (reference) |  |
| College or above | Tertile 2 | 0.39 (0.20 - 0.79) |  |
|  | Tertile 3 | 0.18 (0.10 - 0.34) |  |
|  | Tertile 1 | 1.00 (reference) |  |
| Others | Tertile 2 | 0.79 (0.18 - 3.51) |  |
|  | Tertile 3 | 0.61 (0.08 - 4.47) |  |
| **Marriage** |  |  | 0.7166 |
|  | Tertile 1 | 1.00 (reference) |  |
| Married | Tertile 2 | 0.35 (0.17 - 0.74) |  |
|  | Tertile 3 | 0.20 (0.09 - 0.41) |  |
|  | Tertile 1 | 1.00 (reference) |  |
| Unmarried | Tertile 2 | 0.95 (0.31 - 2.85) |  |
|  | Tertile 3 | 0.43 (0.13 - 1.41) |  |
| **PIR** |  |  | 0.2929 |
|  | Tertile 1 | 1.00 (reference) |  |
| Low | Tertile 2 | 0.96 (0.48 - 1.92) |  |
|  | Tertile 3 | 0.27 (0.12 - 0.59 ) |  |
|  | Tertile 1 | 1.00 (reference) |  |
| High | Tertile 2 | 0.38 (0.14 - 1.02) |  |
|  | Tertile 3 | 0.24 (0.09 - 0.65) |  |

Table S4. Stratified analysis of associations of OBS with Mets in US adult population. Adjusted for confounding factors such as age, gender, race, education, poverty–income ratio. Abbreviation and acronyms: PIR family income-to-poverty ratio.

Table S5. Association between dietary/lifestyle OBS with Mets in US adult population, NHANES 1999–2018 ^1^.

|  | Unadjusted OR (95%CI) | Adjusted OR (95%CI) |
| --- | --- | --- |
| **Dietary OBS** |  |  |
| Mets (OR 95%CI) |  |  |
| Tertile 1 | 1.00 (reference) | 1.00 (reference) |
| Tertile 2 | 0.56 (0.35 - 0.91) | 0.73 (0.40 - 1.31) |
| Tertile 3 | 0.34 (0.23 - 0.49) | 0.51 (0.28 - 0.95) |
| *P* trend | <.0001 | 0.0242 |
| Raised WC (OR 95%CI) |  |  |
| Tertile 1 | 1.00 (reference) | 1.00 (reference) |
| Tertile 2 | 0.84 (0.63 - 1.12) | 0.90 (0.65 - 1.26) |
| Tertile 3 | 0.83 (0.65 - 1.05) | 0.82 (0.60 - 1.13) |
| *P* trend | 0.1245 | 0.212 |
| Raised TG (OR 95%CI) |  |  |
| Tertile 1 | 1.00 (reference) | 1.00 (reference) |
| Tertile 2 | 0.79 (0.62 - 1.01) | 0.83 (0.65 - 1.08) |
| Tertile 3 | 0.66 (0.51 - 0.85) | 0.73 (0.54 - 0.99) |
| *P* trend | 0.002 | 0.0414 |
| Reduced HDL-C (OR 95%CI) |  |  |
| Tertile 1 | 1.00 (reference) | 1.00 (reference) |
| Tertile 2 | 0.85 (0.67 - 1.07) | 0.88 (0.69 - 1.14) |
| Tertile 3 | 0.66 (0.52 - 0.83) | 0.69 (0.49 - 0.96) |
| *P* trend | 0.0007 | 0.0285 |
| Raised BP (OR 95%CI) |  |  |
| Tertile 1 | 1.00 (reference) | 1.00 (reference) |
| Tertile 2 | 0.67 (0.52 - 0.86) | 0.74 (0.54 - 1.02) |
| Tertile 3 | 0.53 (0.42 - 0.69) | 0.65 (0.47 - 0.90) |
| *P* trend | <.0001 | 0.0111 |
| raised FG (OR 95%CI) |  |  |
| Tertile 1 | 1.00 (reference) | 1.00 (reference) |
| Tertile 2 | 0.77 (0.62 - 0.94) | 0.82 (0.64 - 1.04) |
| Tertile 3 | 0.66 (0.55 - 0.79) | 0.78 (0.58 - 1.03) |
| *P* trend | 0.0001 | 0.1013 |
|  |  |  |
| **Lifestyle OBS** |  |  |
| Mets (OR 95%CI) |  |  |
| Tertile 1 | 1.00 (reference) | 1.00 (reference) |
| Tertile 2 | 0.53 (0.28 - 1.02) | 0.54 (0.28 - 1.03) |
| Tertile 3 | 0.17 (0.11 - 0.25) | 0.18 (0.12 - 0.28) |
| *P* trend | <.0001 | <.0001 |
| Raised WC (OR 95%CI) |  |  |
| Tertile 1 | 1.00 (reference) | 1.00 (reference) |
| Tertile 2 | 0.41 (0.31 - 0.53) | 0.38 (0.29 - 0.51) |
| Tertile 3 | 0.17 (0.12 - 0.20) | 0.13 (0.10 - 0.18) |
| *P* trend | <.0001 | <.0001 |
| Raised TG (OR 95%CI) |  |  |
| Tertile 1 | 1.00 (reference) | 1.00 (reference) |
| Tertile 2 | 0.80 (0.60 - 1.07) | 0.82 (0.62 - 1.09) |
| Tertile 3 | 0.49 (0.40 - 0.61) | 0.53 (0.43 - 0.66) |
| *P* trend | <.0001 | <.0001 |
| Reduced HDL-C (OR 95%CI) |  |  |
| Tertile 1 | 1.00 (reference) | 1.00 (reference) |
| Tertile 2 | 0.62 (0.47 - 0.81) | 0.61 (0.46 - 0.81) |
| Tertile 3 | 0.49 (0.40 - 0.59) | 0.49 (0.40 - 0.61) |
| *P* trend | <.0001 | <.0001 |
| Raised BP (OR 95%CI) |  |  |
| Tertile 1 | 1.00 (reference) | 1.00 (reference) |
| Tertile 2 | 0.66 (0.50 - 0.88) | 0.64 (0.48 - 0.88) |
| Tertile 3 | 0.40 (0.31 - 0.51) | 0.35 (0.27 - 0.46) |
| *P* trend | <.0001 | <.0001 |
| raise FG (OR 95%CI) |  |  |
| Tertile 1 | 1.00 (reference) | 1.00 (reference) |
| Tertile 2 | 0.70 (0.56 - 0.87) | 0.69 (0.55 - 0.86) |
| Tertile 3 | 0.43 (0.35 - 0.54) | 0.43 (0.34 - 0.55) |
| *P* trend | <.0001 | <.0001 |
